# Summarizing the Role of Selected Adipokines in Parkinson’s Disease: What Is Known About Leptin, Adiponectin, Resistin, Visfatin, and Progranulin in Neurodegeneration?

**DOI:** 10.3390/molecules30224431

**Published:** 2025-11-16

**Authors:** Jan Milanowski, Marta Pawłowska, Alina Woźniak, Karolina Szewczyk-Golec

**Affiliations:** 1 Students Research Club of Medical Biology, Department of Medical Biology and Biochemistry, Faculty of Medicine, Ludwik Rydygier Collegium Medicum in Bydgoszcz, Nicolaus Copernicus University in Toruń, 24 Karłowicza St., 85-092 Bydgoszcz, Poland; 300672@stud.umk.pl; 2 Department of Medical Biology and Biochemistry, Faculty of Medicine, Ludwik Rydygier Collegium Medicum in Bydgoszcz, Nicolaus Copernicus University in Toruń, 24 Karłowicza St., 85-092 Bydgoszcz, Poland; karosz@cm.umk.pl

**Keywords:** adipokines, Parkinson’s disease, inflammation, oxidative stress

## Abstract

Parkinson’s disease (PD) is the second most common neurodegenerative disease worldwide. It is characterized by the accumulation of α-synuclein, and its symptoms arise from the loss of dopaminergic neurons in the substantia nigra, contributing to the development of both motor (MS) and non-motor (NMS) symptoms. The detailed pathomechanism of the disease progression is unknown, although microglia activation and ongoing neuroinflammation are thought to play key roles. It is known that adipokines have a wide-ranging impact on various processes, including those implicated in PD. We have analyzed a series of studies regarding the significance and involvement of leptin, adiponectin, resistin, visfatin, and progranulin in neurodegeneration. Available evidence suggests that adipokines modulate PD pathology through their effects on inflammation, oxidative stress, or α-synuclein accumulation. Thus, the examined adipokines may serve as potential targets for PD treatment or as biomarkers of disease progression.

## 1. Introduction

Parkinson’s disease (PD) is the second most common neurodegenerative disorder [1]. PD affects approximately 1% of the population over 60 and occurs more frequently in men than women [2,3]. When the onset of the disease occurs between the ages of 21 and 40, it is referred to as Young Onset Parkinson’s Disease (YOPD), constituting approximately 3 to 5% of all PD cases [4]. Several potential etiological factors, including genetic, mitochondrial, immunological, inflammatory, and environmental factors, have been considered for PD, but none have been identified as specific or solely responsible [5,6]. PD, as a synucleinopathy, is characterized by alpha-synuclein accumulation in neurons and glia, forming Lewy bodies [5]. Activation of microglia, which can induce neuroinflammation and, consequently, neurodegeneration, is believed to play a significant role in the pathogenesis of PD [6,7]. PD onset and progression are primarily associated with neuronal loss in the substantia nigra pars compacta (SNc) [8]. Two types of symptoms, including motor symptoms (MS) and non-motor symptoms (NMS), can be distinguished in PD [9]. Core motor symptoms of PD, such as rigidity, bradykinesia, and postural instability, form the basis for diagnosis [10]. Importantly, NMSs often represent prodromal signs of PD, preceding MS by years. Among NMSs, loss of smell and constipation are widespread [9,11]. Treatment of PD should be individualized according to disease stage and symptom profile [12]. Unfortunately, complications such as dyskinesia may develop during treatment with certain medications, reducing the quality of life and necessitating treatment modification [13]. Ongoing research focuses on identifying novel biomarkers, refining current knowledge, and improving early PD diagnosis [14]. Adipokines have a wide-ranging influence on various physiological processes, including those related to PD, such as inflammation and oxidative stress. They may represent potential therapeutic targets or key components of pathogenic pathways involved in PD development, offering new insights into the disease’s fundamental mechanisms [15]. Therefore, the present review aimed to analyze studies investigating the role of adipokines, which appear particularly important in PD, namely leptin, adiponectin, resistin, visfatin, and progranulin.

## 2. Methods

This article is a narrative review summarizing current evidence on the role of adipokines in PD. The literature search was performed in PubMed, Scopus, and Web of Science databases up to May 2025, using the following keywords: Parkinson’s disease, Parkinsonism, adipokine, leptin, adiponectin, resistin, visfatin, NAMPT, and progranulin. Only English-language studies involving human, animal, or in vitro models relevant to PD and adipokine signaling were considered.

Titles and abstracts were screened for relevance, and full texts were reviewed to extract information on study design, population or model, primary outcomes, and proposed mechanisms. Studies focusing solely on metabolic disorders without a PD context were excluded.

Although this review does not follow a systematic PRISMA protocol, key principles of transparent literature synthesis were applied, including explicit search strategy, selection criteria, and critical interpretation of data quality.

Most clinical studies had small sample sizes and heterogeneous populations, and inconsistently controlled for confounders such as body mass index (BMI), diabetes, or medication use. Laboratory assays for adipokines also varied across studies, limiting comparability. Preclinical models provided mechanistic insights but often lacked translational validation. Therefore, conclusions should be interpreted cautiously, as the evidence is low to moderate and largely exploratory.

As a narrative synthesis, this review may be subject to selection bias and incomplete retrieval of relevant literature. The diversity of experimental approaches and measurement methods limits direct comparison across studies. Publication bias, which favors positive findings, cannot be ruled out. Nevertheless, this review integrates data from multiple research domains to provide a holistic overview of adipokine involvement in PD and to identify areas requiring systematic investigation.

## 3. The Role of Adipokines in Parkinson’s Disease

Adipokines are bioactive molecules secreted by adipose tissue that play a crucial role in regulating metabolism, cardiovascular function, and the inflammatory response. They are increasingly recognized for their role in the neuroinflammatory processes associated with PD. These molecules have a significant impact on various physiological processes. In the context of PD, adipokines play a crucial role in elucidating the intricate relationship between systemic metabolic states and neuroinflammation [15,16,17,18].

### 3.1. Leptin

**Figure 1:**
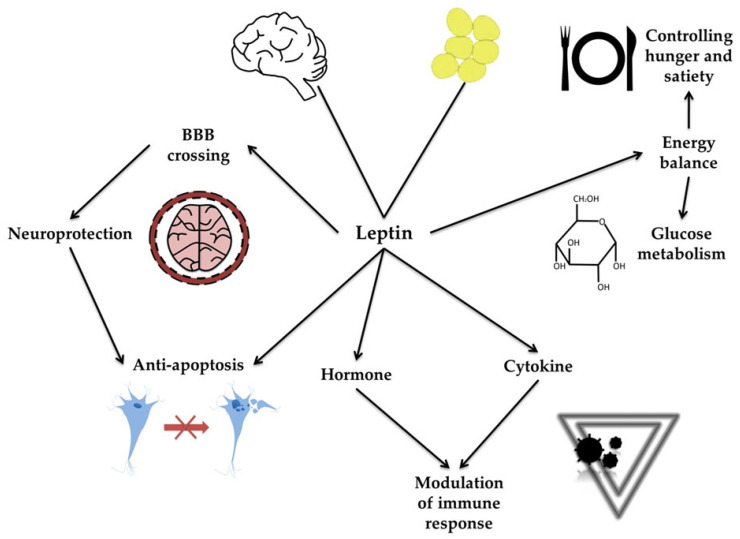
Molecular mechanisms of leptin action. The figure illustrates the molecular mechanisms of leptin action, emphasizing its diverse physiological roles. Leptin, produced primarily by adipose tissue, can cross the blood–brain barrier (BBB) and exert neuroprotective and anti-apoptotic effects in the brain. Acting as both a hormone and a cytokine, leptin modulates immune responses. Through these interconnected pathways, leptin influences metabolic functions, immune regulation, and neuronal survival.

Leptin is an adipokine produced by several tissues, including the brain [16]. Its primary function is to control energy balance, hunger, satiety, and glucose metabolism [19]. Leptin can cross the blood–brain barrier (BBB). Acting as both a hormone and a cytokine, leptin enhances the immune response and exerts anti-apoptotic and neuroprotective effects (see Figure 1) [20]. The improper secretion and function of leptin have been associated with carcinogenesis and obesity and have also been implicated in neurodegeneration and PD [19,20,21,22]. PD progression is often accompanied by significant weight loss. In the study by Fiszer et al. [23], a correlation between weight loss and leptin levels was observed in patients with PD, compared with patients without weight loss and controls. BMI and adipose tissue content also correlated positively with leptin levels in PD patients. However, no correlation was found between leptin and ghrelin, growth hormone (GH), or insulin-like growth factor 1 (IGF-1) levels in any PD group.

Deep-brain stimulation (DBS) is an interesting therapeutic option for patients with PD. It involves the implantation of electrodes connected to a stimulator in brain regions such as the subthalamic nucleus (STN) or the globus pallidus internus (GPi) [24,25]. DBS is generally believed to act by directly stimulating specific brain targets or suppressing pathological activity [26]. In the study by Markaki et al. [27], weight gain after DBS surgery in PD patients during a 6-month follow-up period was correlated with improvements in tremor and bradykinesia in the off-medication phase. In the study by Novakova et al. [28], anthropometric parameters and associated adipokines were assessed in a cohort of STN-DBS patients over 12 months. Leptin levels showed a positive correlation with body weight in this patient group. Similarly, Escamilla-Sevilla et al. [29] investigated the relationship between leptin and STN-DBS in PD, with a focus on weight gain. Over a 6-month follow-up period, surgically treated patients showed a significant increase in leptin levels. Additionally, at the end of the study follow-up, leptin showed a positive correlation with neuropeptide Y. Accordingly, Carrillo et al. [30] reported that in the studied cohort of patients after bilateral DBS, leptin levels significantly increased, reaching values comparable to those observed in the control population.

**Figure 2:**
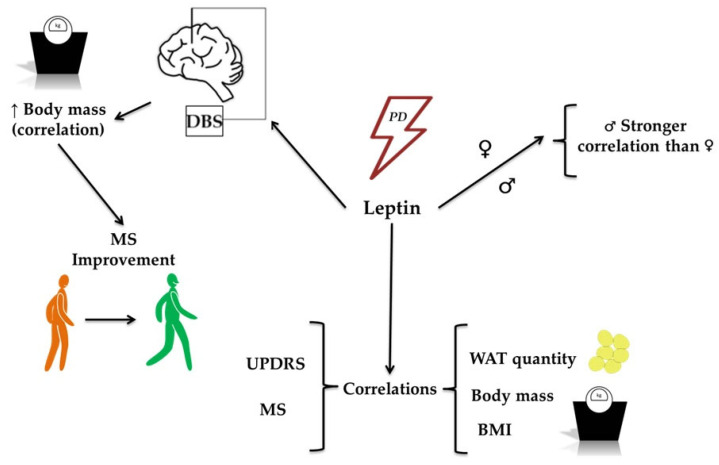
The role of leptin in the development of Parkinson’s Disease (PD). The figure illustrates the role of leptin in PD development, highlighting its associations with clinical and physiological parameters. Leptin levels are associated with PD and show correlations with metrics such as the Unified Parkinson’s Disease Rating Scale (UPDRS) and motor symptoms (MS). Increased body mass is correlated with leptin levels and is associated with improvements in MS, particularly in patients undergoing deep-brain stimulation (DBS). Additional associations include leptin’s relationship with the quantity of white adipose tissue (WAT), body mass, and body mass index (BMI), further reinforcing its role in metabolic and clinical features associated with PD.

Lorefalt et al. [31] observed that leptin levels correlated with body mass in women with newly diagnosed PD before L-DOPA treatment. In line with other studies, leptin levels were lower in men than in women, both in PD patients and in controls. Blood serum leptin concentrations correlated with body fat mass in all examined groups. Significantly, body mass loss in PD patients was associated with lower leptin levels than in patients who did not lose weight. Similarly, Salari et al. [18] observed that leptin levels were statistically significantly lower in PD patients than in healthy controls. However, men had lower leptin levels than women. Moreover, a correlation between disease duration and BMI was observed in PD patients (see Figure 2). In the study by Rocha et al. [32], as expected, higher leptin levels were observed in women than in men. In patients with PD, elevated leptin levels correlated with older age and higher BMI. On the other hand, a study by Kenangil et al. [33] found that leptin levels did not differ between patients with PD and controls. Leptin correlated with body mass, waist circumference, and BMI in PD patients. However, no correlation was found between leptin levels and disease duration or dopamine treatment dosage. In the Bernhardt et al. [34] study, leptin showed a significant correlation with total body fat content, whereas changes in body mass were associated with PD duration.

Lower leptin levels have been reported in PD patients with documented weight loss during disease progression, despite no significant differences in appetite, hunger, satiety, amount of possible food intake, or thirst before meals [35]. At the molecular level, it was demonstrated that bromocriptine, a dopamine agonist used in PD treatment, counteracts diabetogenic effects and inhibits the Janus kinase 2 (JAK2)/phosphorylated signal transducer and activator of transcription 3 (p-STAT3)/suppressor of cytokine signaling 3 (SOCS3) pathway by reducing its two activators, namely interleukin 6 (IL-6) and leptin [36]. Ozdilek et al. [37] did not detect correlations between leptin and clinical PD indicators, suggesting a possible association with dopamine agonist treatment. However, Milanowski et al. [15] demonstrated that leptin concentrations were significantly lower in PD patients than in the control group, with the lowest values observed in PD patients without dyskinesia, suggesting changes in leptin secretion during disease progression. In the study by Nakamura et al. [38], leptin initially showed a correlation with BMI in both PD patients and controls. In PD patients, orthostatic systolic blood pressure changes correlated with baseline and post-tilting leptin levels.

Numerous studies using animal and cell models have explored the links between leptin, neuroinflammation, and PD. 6-hydroxydopamine (6-OHDA) is used to induce PD in animal models [39]. It contributes to the death of dopaminergic nerve cells [40]. Weng et al. [41] investigated the impact of leptin on 6-OHDA-induced cell death in a murine model of PD. Pre-administration of leptin before introducing 6-OHDA reversed its neurotoxic effects in a dose-dependent manner. However, leptin did not protect cells with JAK2 and growth factor receptor-bound protein 2 (GRB2) knockdown. 6-OHDA was found to downregulate leptin production in the substantia nigra of rats [20]. However, hydrogen sulfide (H_2_S) could reverse this effect. Interestingly, blocking the leptin signaling pathway reversed the protective effect of H_2_S against 6-OHDA-induced disturbance of the Warburg effect in the substantia nigra. Jiang et al. [42] conducted an experiment confirming the above findings. They determined that the administration of leptin-OBR, a blocking antibody of leptin receptor (OBR), reversed the effects of NaHS in the substantia nigra of rats exposed to 6-OHDA, elevating caspase-3 activity, increasing B-cell leukemia/lymphoma 2 (Bcl-2)-associated X protein (Bax) expression, and reducing Bcl-2 expression. Additionally, leptin-OBR administration decreased the number of autophagosomes and increased the number of autolysosomes in 6-OHDA-treated rats. In the study by Ujvari et al. [43], the role of neurodegeneration of urocortin 1 (UCN1)-containing cells in the centrally projecting Edinger-Westphal nucleus (EWcp) in mood-related NMS in the rat PD toxic model was investigated. Leptin-saporin toxin, a chemical conjugate of the recombinant mouse leptin and the ribosome-inactivating protein saporin, was administered to eliminate cells that recognize and internalize leptin, thereby inducing cell ablation. This resulted in an increase in anhedonia and signs of anxiety in the rats. However, the procedure did not affect the rats’ motor abilities. Injection led to a decrease in EWcp/UCN1 cells, suggesting neurodegeneration [43].

**Figure 3:**
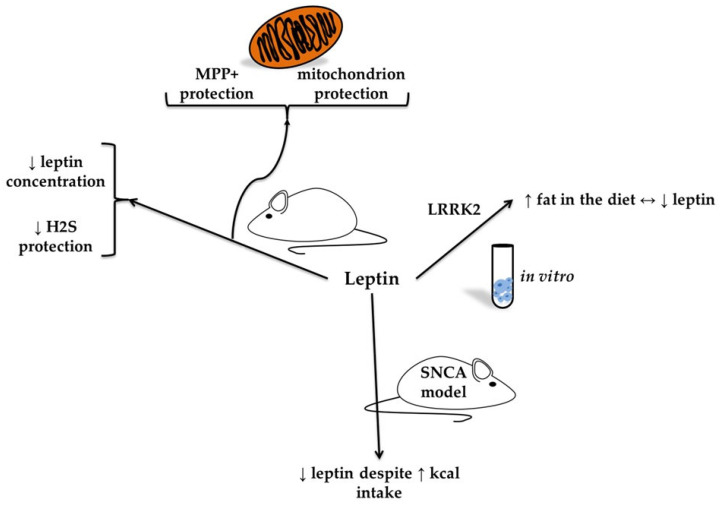
In vitro and animal models of leptin in neurodegeneration (based on [47,48]). The figure presents findings from in vitro and animal models on the role of leptin in neurodegeneration. In models of neural cells exposed to the neurotoxin 1-methyl-4-phenylpyridinium (MPP+), leptin was associated with mitochondrial protection. In the 6-hydroxydopamine (6-OHDA) rodent model of Parkinson’s disease, a reduction in leptin concentration was observed, linked to diminished protective effects of hydrogen sulfide. Additionally, studies in leucine-rich repeat kinase 2 (LRRK2) knockout mice have shown that a high-fat diet reduces leptin levels, suggesting a reciprocal relationship.

A mutation in the gene encoding leucine-rich repeat kinase 2 (LRRK2) is known to cause autosomal-dominant PD, both sporadic and familial [44,45]. It is associated with late-onset disease and phenotypically resembles idiopathic PD (IPD). Kawakami et al. [46] investigated the relationship between LRRK2 and autosomal dominant PD, as well as the causative gene product of the locus for Parkinson’s disease 8 (PARK8). Wild-type (WT) and Lrrk2 exon 41 knockout (KO) mice were exposed to a standard or high-fat diet for 5 months. Leptin levels tended to be lower in KO mice on a high-fat diet than in WT mice, but this difference did not reach statistical significance. In the experiment described by Rothman et al. [47], mice with the T53A mutation in the human alpha-synuclein gene (SNCA) were hypoleptinemic compared to WT mice. A high-calorie diet administered for 12 weeks resulted in a significant increase in circulating leptin levels in WT mice. In contrast, SNCA mice exhibited significantly lower leptin levels under the same dietary conditions (see Figure 3, see Table 1).

Differentiated and undifferentiated human neuroblastoma HS-SY5Y cells were used to assess the impact of leptin on cell survival following exposure to methyl-4-phenylpyridinium (MPP+), a neurotoxin used in cellular models of PD [48]. Leptin alone induced the proliferation of undifferentiated cells. Conversely, MPP+ exerted precisely the opposite effect, decreasing proliferation by 23% compared to the control. The combined application of leptin and MPP+ resulted in proliferation and survival levels similar to those of untreated cells. Leptin blocked the depolarization of the mitochondrial membrane induced by MPP+. However, this effect was not observed in cells knocked down for uncoupling protein 2 (UCP2). Leptin maintained adenosine 5′-triphosphate (ATP) levels similar to those in MPP+-untreated cells. Furthermore, leptin increased mRNA and protein levels of UCP2 and uncoupling protein 4 (UCP4). Interestingly, leptin did not block an increase in reactive oxygen species (ROS) production induced by MPP+ [48].

These studies present a complex but convincing picture of leptin’s role in PD, supporting its involvement in neuroinflammatory and metabolic pathways. Table 1 summarizes human, animal, and in vitro studies of leptin in PD. The results of studies on patients with PD do not clearly determine the direction of leptin changes in the course of the disease. However, these changes may reflect alterations in body mass, a common feature of PD. Studies conducted in animal and cell models show great promise for neuroprotection, but their translation to clinical practice appears distant and requires numerous additional experiments. Undoubtedly, inconsistencies in clinical data, mechanistic uncertainty, and a lack of translational application limit the ability to draw definitive conclusions. More integrated, longitudinal, and mechanistically driven studies are needed to determine whether leptin is a causative factor, a compensatory response, or simply a metabolic byproduct in the PD cascade.

It is worth emphasizing that leptin exhibits multifaceted neuroprotective properties in experimental PD models, including antioxidative, anti-apoptotic, and anti-inflammatory actions. However, clinical data remain contradictory. Some studies show reduced serum leptin levels in PD, while others report elevated levels linked to BMI or dopaminergic therapy. Methodological differences, disease heterogeneity, and confounding by metabolic factors likely contribute to inconsistent findings. Overall, leptin may act as a compensatory neuroprotective signal rather than a reliable biomarker of disease severity.

### 3.2. Adiponectin

**Figure 4:**
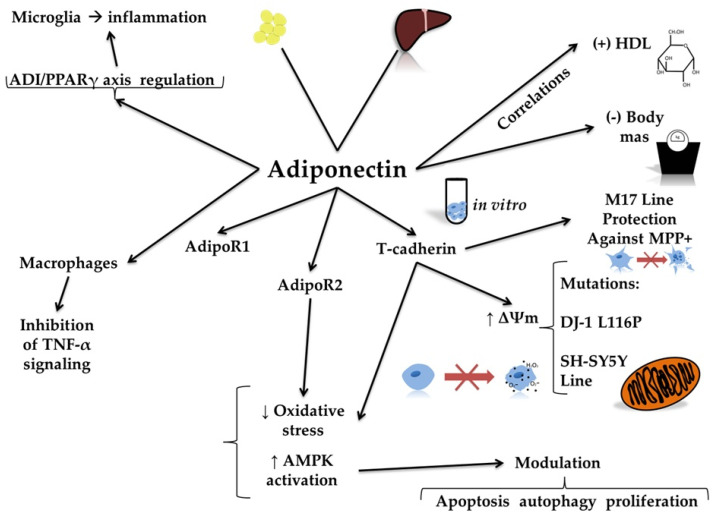
Molecular mechanisms of adiponectin action. The figure illustrates the molecular mechanisms of adiponectin action, highlighting its diverse physiological and cellular effects. Adiponectin acts through its receptors, namely AdipoR1 and AdipoR2. Adiponectin results in decreased oxidative stress and increased activity of adenosine 5′-monophosphate-activated protein kinase (AMPK). Adiponectin also interacts with T-cadherin, enhancing mitochondrial membrane potential (∆Ψm) and protecting cell lines, such as the human neuroblastoma M17 line, against mitochondrial toxins like 1-methyl-4-phenylpyridinium (MPP+). This protective effect is especially relevant in the case of DJ-1 L116P mutations in human neuroblastoma SH-SY5Y cells. Additionally, adiponectin regulates immune responses. It inhibits tumor necrosis factor alpha (TNF-α) signaling in macrophages and reduces inflammation in microglia through the adiponectin (ADI)/peroxisome proliferator-activated receptor gamma (PPARγ) axis. At the systemic level, adiponectin shows a positive correlation with high-density lipoprotein (HDL) levels and a negative correlation with body mass index.

Adiponectin, one of the best-known adipokines, is secreted mainly by white adipose tissue but also, at lower levels, by hepatocytes, monocytes, and placental cells [49,50]. Adiponectin isoforms can modulate gene expression patterns implicated in the pathogenesis of several diseases [51]. Adiponectin exists in three forms, characterized by low-, medium-, and high-molecular-weight (HMW) [52,53]. The HMW form is a multimer (12- to 18-mer) that acts as an anti-inflammatory agent, providing protective effects on the cardiovascular system and exhibiting antidiabetic effects [51,54]. The intermediate-molecular-weight form primarily regulates lipid metabolism and energy balance [55]. Three adiponectin receptors, AdipoR1, AdipoR2, and T-cadherin, are expressed in various tissues, including the brain, pancreas, heart, liver, and muscles. Adiponectin exerts anti-inflammatory effects by inhibiting tumor necrosis factor alpha (TNF-alpha) signaling and reducing pro-inflammatory cytokine activity in macrophages [56,57]. Furthermore, this adipokine ameliorates oxidative stress by engaging AdipoR2 and activating adenosine monophosphate-activated protein kinase (AMPK), thereby modulating autophagy, apoptosis, and proliferation (see Figure 4) [56,58].

Several studies have investigated the association between adiponectin and neurodegeneration, including PD (Table 2) [56,59,60]. Cassani et al. [58] found a positive correlation between serum adiponectin levels and high-density lipoprotein (HDL) concentration in PD patients. Thus, low adiponectin levels may be associated with an increased risk of cardiovascular events in patients, which should be considered during therapy [61,62,63]. Additionally, an inverse correlation was detected between adiponectin and waist circumference, body mass, fasting glucose concentrations, and triglyceride levels in PD patients (see Figure 4) [58].

It has been demonstrated that the adiponectin/peroxisome proliferator-activated receptor gamma (PPARγ) axis is crucial for mitigating cognitive impairments resulting from chronic hypoperfusion. This mechanism depends on the regulation of microglial activity and inflammation and has been described in vascular cognitive impairment and dementia [64]. Furthermore, agonists of AdipoR1 and AdipoR2 have been reported to enhance cell survival and reduce oxidative stress in rat retinal ganglion cells treated with glutamate [65]. Analogous effects were observed in traumatic brain injury [66]. Adiponectin has also been shown to be significant in Alzheimer’s disease (AD), as it stimulates insulin sensitivity, exhibits anti-inflammatory and antioxidant effects, and protects against the production of amyloid beta and Tau protein phosphorylation caused by decreased cerebral insulin signaling activities [67]. *PARK7* encodes DJ-1, also known as the Parkinson’s disease protein 7 (PARK7) [68]. The proper functioning of this protein involves antioxidant activity, which is part of neuroprotective mechanisms [68,69]. Additionally, PARK7 regulates apoptosis, mitochondrial function, and dopamine homeostasis [69,70]. Li et al. [71] proposed that adiponectin exhibits protective activity against the neurotoxicity of the leucine 166 proline (L166P) familial mutation of DJ-1. The researchers based their findings on studies conducted on human neuroblastoma M17 cells. The DJ-1 L166P mutation exhibits multifaceted toxicity, inducing ROS and nitric oxide (NO) production while decreasing mitochondrial membrane potential [68,72]. In vitro studies have demonstrated that supplying adiponectin at appropriate doses reduced the negative impact of the mutation on the transfected cells. The results showed that AdipoR2 levels in the transfected cells were lower than in the positive control, Michigan Cancer Foundation-7 (MCF-7) cells. In PD modeling, MPP+ is utilized as a marker of oxidative stress, with a particular affinity to the substantia nigra [73,74]. Previous studies by Jung et al. [60] supported the above findings regarding adiponectin’s attenuation of MPP+ action. As reported by Sekyiama et al. [54], histological examination of collected brain samples revealed anti-adiponectin antibody reactivity in Lewy bodies, mainly observed in PD patients with phosphorylated alpha-synuclein. Adiponectin inhibited neurodegeneration in B103 neuroblastoma cells, resulting in a significant reduction in alpha-synuclein levels. In older mice treated with globular adiponectin, improvements in body mass and a slowing of MS progression were observed compared with controls [54]. However, adiponectin treatment initiated in older mice with more advanced MS did not improve outcomes. In the brain samples of transgenic mice treated with adiponectin, a decrease in the levels of guanosine 5′-monophosphate (GMP) and inosine 5′-monophosphate (IMP), both of which stimulate alpha-synuclein aggregation, was observed, indicating a positive effect of adiponectin [75]. In a rodent model of PD, intranasal (i.n.) administration of L-DOPA and the adiponectin receptor agonist (AdipoRon) significantly improved locomotor and memory performance in rats with 6-OHDA-induced lesions [75]. AdipoRon markedly reduced ROS levels and increased total antioxidant capacity (TAC) in the hippocampus of rats with unilateral 6-OHDA injections. Additionally, a significant increase in superoxide dismutase (SOD) activity was observed compared to untreated PD animals. In contrast, L-DOPA alone did not produce any noticeable changes in the PD group. In the study by Park et al. [76], mice treated with N-methyl-4-phenyl-1,2,3,6-tetrahydropyridine (MPTP), a widely used neurotoxin to induce PD-like symptoms in animal models, and mice expressing human α-synuclein exhibited a significant reduction in motor performance. Interestingly, mice treated with phytochemical osmotin, a homolog of adiponectin, showed improvement in tests of neuromuscular strength, suggesting alleviation of MS.

Considering the changes in adiponectin in PD, several studies have been conducted. Rocha et al. [32] did not find a statistically significant difference in adiponectin concentration between PD patients and the control group [73]. Accordingly, Milanowski et al. [15] did not detect a statistically significant difference in adiponectin concentration between patients with PD and healthy controls, or between PD patients with and without dyskinesia. In the study by Barichella et al. [21], adiponectin was identified as a marker of improved cardioprotective factors following sleeve gastrectomy in morbidly obese patients with PD. Interestingly, adiponectin levels were significantly higher in patients with PD compared to those with progressive supranuclear palsy (PSP) [77]. Moreover, a positive correlation was observed between adiponectin and both HDL-C and triglyceride levels. Compared to healthy controls, PD patients exhibited elevated adiponectin concentrations. Carrillo et al. [26] reported a significant increase in adiponectin levels in PD patients treated with L-DOPA, which was reversed following DBS treatment.

It should be noted that, in the available literature, inconsistent adiponectin levels across human studies can be observed. While some studies suggest elevated adiponectin levels in PD patients compared with healthy controls or PSP patients, others report no significant differences between healthy subjects and PD patients, or between PD subgroups with and without dyskinesia. This variability may be due to small sample sizes, heterogeneous patient populations, confounding metabolic conditions (e.g., obesity, diabetes), or different assay sensitivity. These inconsistencies limit the current utility of adiponectin as a biomarker or therapeutic target and highlight the need for standardized, long-term human studies.

The neuroprotective effects described, such as attenuation of alpha-synuclein aggregation, reduction in ROS generation, and improvement of behavioral symptoms, are impressive but not disease-specific. These effects are also relevant to AD, traumatic brain injury, and vascular cognitive impairment, suggesting that adiponectin acts as a general neuroprotective agent rather than a PD-specific modulator. These findings prompt the question of whether adiponectin targets PD-specific mechanisms, such as α-synuclein aggregation, or acts via broader metabolic and anti-inflammatory pathways.

While adiponectin shows promise in modulating inflammation, oxidative stress, and potentially α-synuclein toxicity in PD, the current evidence is mainly preclinical and correlative in human studies. The heterogeneity in findings across populations and experimental models necessitates caution before proposing adiponectin as a definitive therapeutic target or biomarker. Future research should focus on longitudinal human studies, receptor-specific agonist trials, and the impact of existing PD treatments, such as L-DOPA and DBS, on the adipokine profile. This will be critical in determining whether adiponectin has a causal role in neurodegeneration or is merely a marker of systemic metabolic and inflammatory states.

In summary, adiponectin exerts anti-inflammatory and metabolic regulatory effects, attenuating oxidative stress and improving mitochondrial biogenesis in experimental PD models. Human data are inconsistent, with both elevated and reduced circulating levels reported, often influenced by sex, adiposity, and comorbid metabolic conditions. While mechanistic studies support a neuroprotective potential, clinical validation is limited. Adiponectin likely reflects systemic metabolic adaptation rather than a direct correlate of PD progression.

**Table 2:** Human and animal studies on adiponectin in Parkinson’s disease (PD).

Study Characteristics	Results	References
30 PD subjects 28 morbidly obese subjects 33 normal-weight subjects	Adiponectin correlated with HDL in PD patients; it showed inverse correlations with waist circumference, body mass, fasting glucose concentrations, and triglycerides.	Cassani et al. [59]
40 PD subjects 25 control subjects	The concentrations of adiponectin, resistin, and leptin did not correlate with the clinical data of patients with PD.	Rocha et al. [32]
44 PD subjects: 20 No dyskinesia 24 With dyskinesia 20 control subjects	Adiponectin levels did not differ between the study groups.	Milanowski et al. [15]
47 PD subjects: 15 bilateral DBS 16 on L-DOPA treatment 16 no L-DOPA treatment 16 controls	Adiponectin levels increased with L-DOPA treatment and reversed after DBS.	Carrillo et al. [30]
54 PD subjects: 8 untreated 14 with dyskinesia 17 MSA-P 25 PSP 23 control group	Higher adiponectin levels were observed in PD compared with PSP and controls, and adiponectin levels correlated positively with HDL-C and triglycerides.	Kataoka et al. [77]
Neuroblastoma M17 cell-line with DJ-1 L166P mutation compared to control MCF-7 cells	Adiponectin reduced MPP+ in mutant cells.	Li et al. [71]
SH-SY5Y cell line treated with MPTP, measured SOD and caspase-3, intracellular H_2_O_2_, Bcl-2, Bax, cytochrome c and performed fluorescent microscopy	Adiponectin attenuated MPP+ by reducing ROS (SOD and catalase activity down), blocked nuclear morphological changes, and maintained Bax mRNA and Bcl-2 at control levels. Adiponectin inhibited caspase-3 expression.	Jung et al. [60]
Brain tissue from PD, DLB patients, and controls examined; B103 neuroblastoma cells producing alpha-synuclein treated with adiponectin; transgenic mice expressing wild-type human alpha-synuclein treated with gAPN every 3 days for 2 months; 5-month-old mice also treated with i.n. gAPN.	Anti-adiponectin reactivity found in Lewy bodies in PD. Adiponectin reduced alpha-synuclein in B103 cells. Globular adiponectin improved body mass and slowed motor symptoms in older mice. No improvement in advanced MS. Adiponectin reduced GMP/IMP, inhibiting alpha-synuclein aggregation.	Sekyiama et al. [54]
Rats treated with 6-OHDA PD-induced in 5 groups: -treated with DMSO -p.o. L-DOPA 10 mg/kg -i.n. AdipoRon at 0.1 μg in 10 μL/rat -i.n. AdipoRon at 1 μg in 10 μL/rat -i.n. AdipoRon at 10 μg in 10 μL/rat.	AdipoRon + L-Dopa improved locomotion and memory. AdipoRon reduced ROS and increased TAC and SOD in the hippocampus. L-DOPA alone was ineffective.	Alimohammadi et al. [75]
SH-SY5Y cells, BV-2 cells, mHippoE-14 cells cultured and treated with MPP+; The C57BL/6J mouse groups included: control, MPTP, and MPTP with osmotin. The transgenic C57BL/6 mice were divided into three groups: WT, α-syn, and α-syn + osmotin.	MPTP-treated and human α-syn-expressing mice exhibited reduced motor performance. Osmotin-treated mice showed improved neuromuscular strength, alleviating motor symptoms. AdipoR1 knockout cells demonstrated that osmotin did not activate AMPK, confirming receptor dependence. Osmotin might enhance α-synuclein clearance.	Park et al. [76]

Abbreviations: Bax—Bcl-2-like protein 4, Bcl-2—B-cell lymphoma 2 protein, DJ-1—Parkinson’s disease protein 7, HDL—high-density lipoprotein, L166P—leucine 166 proline mutation, MPP+—1-methyl-4-phenylpyridinium, MPTP—N-methyl-4-phenyl-1,2,3,6-tetrahydropyridine, ROS—reactive oxygen species, SH-SY5Y cells—human neuroblastoma cells, SOD—superoxide dismutase.

### 3.3. Resistin

**Figure 5:**
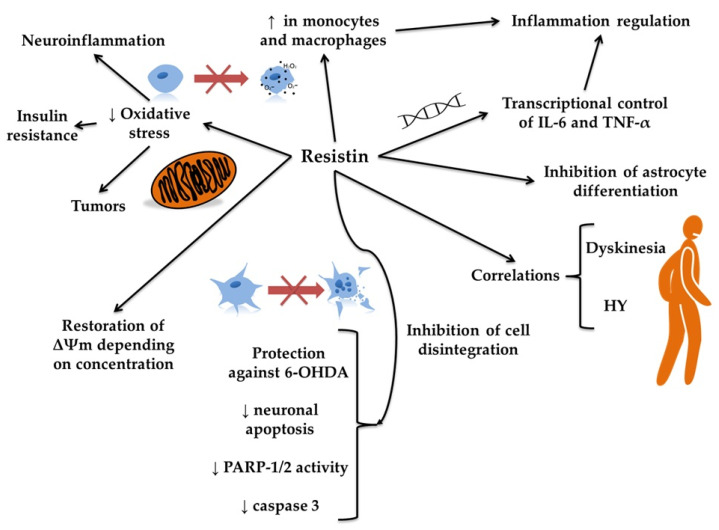
The putative role of resistin in Parkinson’s disease. The figure illustrates the proposed role of resistin in Parkinson’s disease by depicting its involvement in multiple cellular and molecular pathways. Resistin appears to influence inflammation by transcriptionally regulating pro-inflammatory cytokines such as interleukin 6 (IL-6) and tumor necrosis factor alpha (TNF-α), thereby regulating inflammation and inhibiting astrocyte differentiation. Increased expression of resistin in monocytes and macrophages might modify neuroinflammation and the intensity of oxidative stress. This, in turn, may protect against oxidative stress-induced cell damage and support mitochondrial function by restoring membrane potential (Δψm), depending on the resistin concentration. Furthermore, resistin may protect neurons against 6-hydroxydopamine (6-OHDA)-induced toxicity by reducing neuronal apoptosis and decreasing the activity of poly(ADP-ribose) polymerase-1 and 2 (PARP-1/2) and caspase 3, contributing to the inhibition of cell disintegration. These cellular effects of resistin correlate with clinical features of Parkinson’s disease, such as dyskinesia and Hoehn and Yahr (HY) stage progression, suggesting that resistin may be implicated in both the pathology and symptomatology of the disease.

Resistin is an adipokine strongly associated with inflammation and inflammation-related diseases [78]. It is involved in regulating the transcription of pro-inflammatory cytokines, such as IL-6 and TNF-alpha [79]. High concentrations of resistin are detected in monocytes and macrophages [80]. In addition to its pro-inflammatory activity, resistin promotes oxidative stress and has been implicated in tumor development, insulin resistance, and neurodegeneration (see Figure 5) [15,16,81]. Aziz et al. [82] investigated the secretion and concentration of resistin in patients with PD over a 24 h period. However, no significant changes were detected. Xiaoying et al. [83] investigated the impact of resistin on the proliferation and differentiation of neural stem cells (Table 3). After resistin treatment, they observed a decrease in the glial fibrillary acidic protein (GFAP)+/bromodeoxyuridine (BrdU)+ cells, indicating that resistin may inhibit astrocyte differentiation. Lu et al. [84] found that resistin protected against morphological damage in hybrid dopaminergic cells derived from the murine neuroblastoma-glioma MES23.5 induced by 6-OHDA. Using annexin V staining, the authors found that resistin protected cells against 6-OHDA-induced apoptosis, as evidenced by reduced nuclear condensation, fragmentation, and cell size reduction compared with the control. Additionally, resistin reduced the response to annexin V in early apoptosis. The treatment with 6-OHDA results in a decrease in Bcl-2 expression and a significant increase in Bax expression. Pretreatment with resistin, in a concentration-dependent manner, reversed the Bax/Bcl-2 ratio [84]. The expression of activated poly(ADP-ribose) polymerase-1 and 2 (PARP-1/2) and cleaved caspase-3 also increases after exposure to 6-OHDA. Pretreatment with resistin, in a concentration-dependent manner, reversed the effects of 6-OHDA by reducing PARP-1/2 and caspase-3 levels. Moreover, resistin reduced intracellular ROS levels by inhibiting the production of superoxide anion (O_2_^•−^) and H_2_O_2_ induced by 6-OHDA [84]. It is suggested that mitochondrial membrane potential (Δψm) is one of the factors determining cell survival and proper functioning; thus, measuring Δψm can provide information about the state of the examined cells [85]. It was found that resistin can restore impaired mitochondrial function. Pretreatment with resistin significantly reversed the decrease in Δψm in a concentration-dependent manner [84]. Interestingly, resistin applied to 6-OHDA-untreated cells had no visible effect on Δψm. In the experiment, resistin also increased heat shock cognate protein 73 (Hsc73) expression in a concentration and time-dependent manner and significantly upregulated heme oxygenase-1 (HO-1) expression. Pretreatment with zinc protoporphyrin IX (ZnPPIX) effectively reversed the protective effect of resistin. Conversely, pretreatment with 3-(1,3-benzodioxol-5-ylmethylene)-2-oxo-1-pyrrolidinecarboxaldehyde (KNK 437, heat shock protein inhibitor I) also reversed the protective effect of resistin [84]. The antiapoptotic and antioxidant effects of resistin described above suggest that this adipokine may provide cytoprotection to dopaminergic neurons under conditions of oxidative stress. These results challenge the conventional view of resistin as solely pro-inflammatory and indicate that its effects may be context-dependent.

Studies on resistin levels and the severity of PD are still inconclusive. In the study by Milanowski et al. [15], a correlation was observed between resistin levels and PD severity (Table 3). The level of resistin in the dyskinetic group was significantly higher than in the non-dyskinetic group. The resistin concentration in the advanced stage was approximately 2.7 times higher than in the non-dyskinetic patients. Additionally, resistin levels positively correlated with the Hoehn and Yahr scale [15]. This suggests that resistin may serve as a biomarker of PD severity, particularly in advanced stages. However, this finding must be validated in larger cohorts. In contrast, Rocha et al. [32] found no correlations between resistin and the clinical state or parameters of PD patients.

The relationship between resistin and PD remains poorly defined and requires further investigation. While cell-based findings suggest a neuroprotective potential, human studies are inconclusive and inconsistent. Additionally, resistin’s well-established pro-inflammatory reputation complicates the interpretation of the studies described. Resistin might be viewed as a modulator whose effects may be context-dependent, influenced by the cellular environment, systemic health, and the stage of the disease. Resistin appears to be a promising yet unvalidated target in the landscape of PD neuroinflammation research.

It is worth noting that resistin has primarily been recognized as a pro-inflammatory adipokine that stimulates microglial activation and oxidative stress in cellular models. Clinical studies report higher serum resistin levels in PD compared with controls, suggesting systemic inflammatory involvement. Nevertheless, results are not universal and may depend on coexisting metabolic syndromes. Current evidence supports the view that resistin is a peripheral marker of low-grade inflammation that may amplify PD pathology.

### 3.4. Visfatin

**Figure 6:**
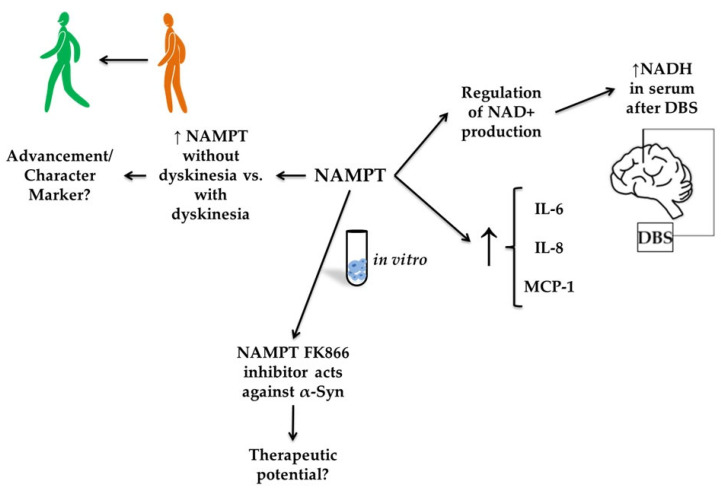
The putative role of visfatin in Parkinson’s disease (PD). The figure illustrates the proposed role of visfatin (nicotinamide phosphoribosyltransferase, NAMPT) in Parkinson’s disease, highlighting its regulatory and potential therapeutic functions. NAMPT expression appears elevated in PD patients without dyskinesia compared with those with dyskinesia, suggesting it may be a marker of disease progression or symptom characterization. In vitro, NAMPT regulates nicotinamide adenine dinucleotide (NAD^+^) production and is associated with increased levels of inflammatory cytokines, including interleukin-6 (IL-6), interleukin-8 (IL-8), and monocyte chemoattractant protein-1 (MCP-1). Furthermore, serum NADH levels increased following deep-brain stimulation (DBS), suggesting a link between NAMPT activity and therapeutic outcomes.

Visfatin (nicotinamide phosphoribosyltransferase, NAMPT) is an adipokine with diverse metabolic and immunomodulatory effects. Initially discovered as a pre-B-cell colony-enhancing factor, it exists in intracellular and extracellular forms [86]. It plays a role in regulating nicotinamide adenine dinucleotide (NAD^+^) production [87]. Extracellularly, visfatin functions via hormonal communication [88]. Studies have demonstrated that NAMPT increases IL-6 and interleukin 8 (IL-8) levels, as well as upregulates monocyte chemoattractant protein-1 (MCP-1) [86]. It is particularly associated with metabolic and cardiovascular diseases (see Figure 6) [89,90,91].

Ostrakhovitch et al. [92] investigated a group of patients undergoing DBS surgery for PD and essential tremor (ET). They demonstrated that serum levels of NAMPT were significantly higher in patients with PD than in those with ET. However, no significant difference was observed in cerebrospinal fluid (CSF) (Table 4). This also implied a higher level of NADH, resulting from increased NAMPT activity in the serum of PD patients compared to ET patients. Increased NAMPT levels in PD may reflect a compensatory response to neurodegeneration, given the established role of NAD^+^ depletion in PD pathogenesis. The fact that NAMPT was elevated in serum, but not in CSF, may suggest that the elevated levels indicate systemic metabolic stress rather than a central neural system (CNS)-specific mechanism. Milanowski et al. [16] evaluated NAMPT in patients with PD, dividing them into groups with and without dyskinesia and comparing them with healthy controls. A statistically significant decrease in NAMPT levels was demonstrated in patients with dyskinesia compared to the non-dyskinetic group. In a study by Parsons et al. [93], post-mortem examinations of PD patients were conducted to assess levels of NAMPT and nicotinamide mononucleotide adenylyl transferase (NMNAT) in the caudate nucleus, in relation to the amount of monomeric alpha-synuclein. It was found that FK866, an inhibitor of NAMPT, may protect neurons against alpha-synuclein expression in dopaminergic (DA) neuron-like SH-SY5Y cells [93]. It highlights a potentially paradoxical role: while NAMPT supports NAD^+^ production, excessive or unregulated activity may promote neuroinflammatory cascades or metabolic dysfunction, especially when alpha-synuclein is overexpressed. The mRNA for NAMPT, one of the markers investigated by Santiago et al. [94], was 69% accurate in distinguishing progressive supranuclear palsy from healthy controls. No such conclusions were drawn for PD and atypical Parkinsonian disorders.

The role of visfatin in PD remains a promising yet insufficiently characterized research area. Current evidence supports that NAMPT functions as a peripheral metabolic and inflammatory signal that may intersect with neurodegenerative pathways, especially those involving mitochondrial stress and alpha-synuclein toxicity. However, inconsistencies in sample sources—such as differences between serum and CSF measurements—along with unclear mechanisms of action (pro- or anti-inflammatory) and a lack of longitudinal data continue to limit its clinical applicability. To advance understanding in this area, future studies should clarify the distinct roles of intracellular versus extracellular NAMPT in neuronal and glial cells, track NAMPT levels throughout the progression of PD and during treatment, and explore the use of NAMPT in combination with other adipokines as potential biomarkers of systemic inflammation in PD. Additionally, research employing animal models with NAMPT modulation will be essential to determine its causal role in neurodegeneration and neuroinflammation. Until these questions are addressed, NAMPT remains a promising yet equivocal component within the broader PD-adipokine-neuroinflammation framework.

Undoubtedly, visfatin supports NAD^+^ biosynthesis and regulates mitochondrial homeostasis, processes disrupted in PD. Experimental studies show that visfatin enhances neuronal survival by activating sirtuin 1 (SIRT1) and antioxidant pathways. Clinical data are scarce and inconsistent, and there is limited evidence on circulating NAMPT levels in PD patients. Future research should clarify whether modulation of the NAD^+^/SIRT1 axis through NAMPT represents a viable neuroprotective strategy.

### 3.5. Progranulin

**Figure 7:**
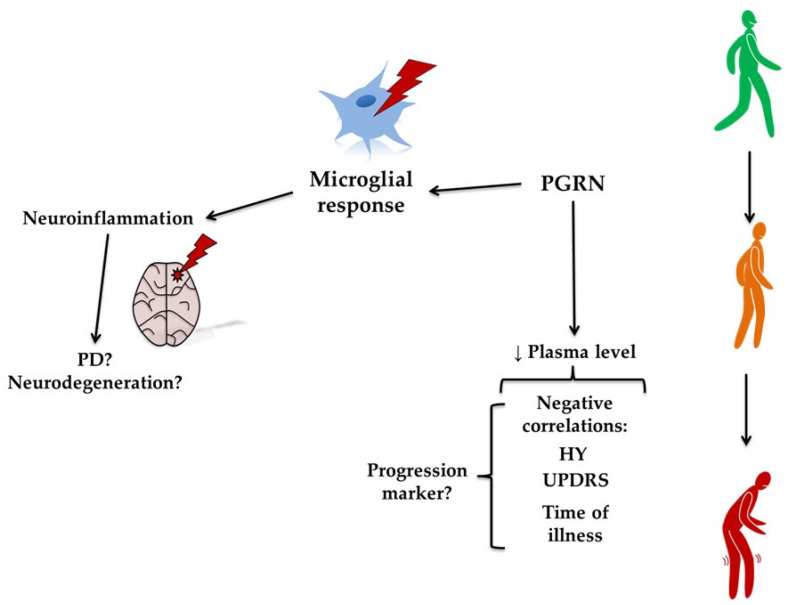
The putative role of progranulin (PGRN) in Parkinson’s disease (PD). The figure illustrates the proposed role of PGRN in PD. A reduction in plasma PGRN levels is negatively correlated with clinical measures, such as the Hoehn and Yahr (HY) stage, the Unified Parkinson’s Disease Rating Scale (UPDRS), and disease duration, suggesting that PGRN could serve as a marker of disease progression. PGRN influences the microglial response, contributing to neuroinflammation. This progression from reduced PGRN to increased inflammation may underlie the worsening motor symptoms observed in PD.

**Figure 8:**
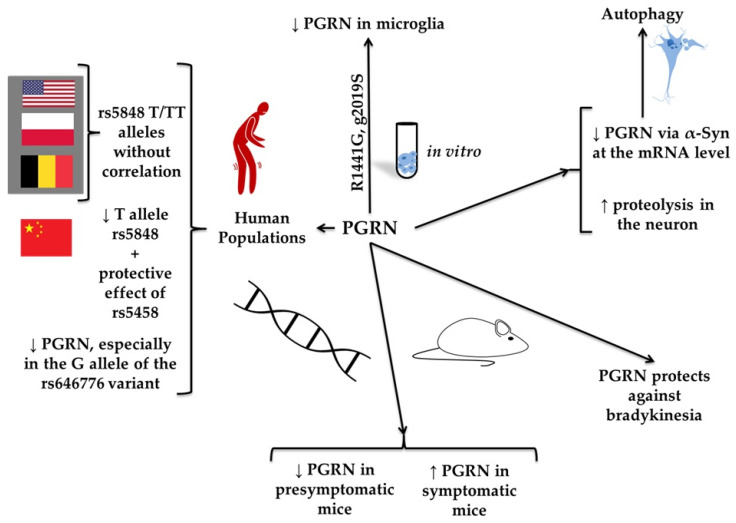
Polymorphism of genes associated with progranulin (PGRN) and Parkinson’s disease (PD). The figure depicts the role of genetic polymorphisms in the PGRN gene and their association with PD. It has been found that in various human populations, such as those from the United States, Poland, and Belgium, the rs5848 T/TT alleles do not correlate with PD. However, the Chinese population was described to have a lower frequency of the rs5848 T allele, along with a protective effect of the rs5458 variant. Additionally, decreased PGRN expression has been observed, particularly in individuals carrying the G allele of rs646776. In vitro studies demonstrated reduced PGRN levels in microglia, particularly under mutations such as R1441G and G2019S. It affected autophagy by decreasing PGRN mRNA levels, involving α-synuclein (α-Syn), and by increasing proteolysis in neurons. In mouse models, PGRN levels were reduced in both the presymptomatic and symptomatic stages of PD, suggesting that PGRN plays a protective role against bradykinesia.

Progranulin (PGRN) is a protein that consists of 7.5 repeated domains and is proteolytically cleaved into granulins by lysosomal proteases [55]. PGRN functions as a growth factor essential for CNS integrity and immune regulation [95]. It also acts as a neuroimmune-modulatory and neuroprotective agent [96]. PGRN may undergo proteolytic cleavage, yielding granulins (GRN) A-G, which exhibit functional properties distinct from the full-length precursor. Dysregulation of GRN processing and its activity may contribute to the neurodegeneration [97,98]. Dysfunction due to PGRN deficiency can lead to an improper microglial response and neuroinflammation, ultimately contributing to neurodegeneration, including the pathogenesis of PD (see Figure 7) [96,99]. PGRN haploinsufficiency is a leading cause of frontotemporal lobar degeneration (FTLD) and lysosomal storage disorders [100]. Studies in the US and Polish populations found no correlation between the GRN rs5848 T allele and PD [101]. The rs5848 TT genotype was comparable between patients with PD and control subjects. Similarly, no association was found between PD and PGRN gene mutations in the Belgian population [102]. Serum GRN levels were significantly lower in Belgian PD patients. There was no correlation between the age of symptom onset and GRN levels. Interestingly, in the rs5848 C/T mutation group, the onset was, on average, 3.5 years earlier than in the C/C variant group. No differences in serum GRN levels were detected between patients with different genotypes for sortilin-1 (SORT1) and prosaposin (PSAP). Additionally, a significant effect of the G4C2 repeat size in the C9orf72 gene on the age of symptom onset was identified. Heterozygous patients with an intermediate repeat length had symptoms, on average, 3.8 years earlier than patients with two short alleles [103]. According to a study on the Belgian population, null mutations are rare in patients with PD, and the clinical diagnosis of PD in patients carrying null mutations in the PGRN gene is most likely due to etiological heterogeneity rather than PGRN haploinsufficiency [104]. The study by Chang et al. [105] demonstrated that the GRN rs5848 single-nucleotide polymorphism (SNP) influenced PD susceptibility in the Taiwanese population. A higher prevalence was observed in individuals with the TT genotype, particularly females. This observation contrasts with the findings of Jasinska-Myga et al. [101], who investigated the US and Polish populations and reported differences in PD risk associated with the GRN rs5848 variant. In the study by Chen et al. [106], the minor allele T of the GRN rs5848 gene was found to be significantly less common in patients with PD compared to controls. The researchers also reported that the GRN rs5458 variant may lower the risk of PD in the Chinese population (see Figure 8).

Mutations in the GRN gene can manifest as Parkinsonism, which clinically resembles IPD. Postural tremor has been associated with GRN mutation within PD [107]. A family demonstrating intrafamilial clinical heterogeneity with PGRN gene deletions was described [108]. Different diagnoses were made within the family, and the clinical presentation was heterogeneous. Parkinsonism in the course of FTLD or PD was not ruled out. Subcortical atrophy ipsilateral to the dominant Parkinsonian syndrome was detected post-mortem. PGRN levels were significantly reduced in the conditioned medium of mouse fibroblasts with the LRRK2(R1441G) mutation, leukocytes, and microglia [109]. In contrast, levels of pro-inflammatory factors, such as interleukin 1β (IL-1β) and keratinocyte-derived chemokine, were significantly increased. Decreased PGRN levels were also detected in human fibroblasts isolated from carriers of the LRRK2 (G2019S) mutation, while mitochondrial function remained unaffected [109]. Additionally, levels of matrix metalloproteinase 2 (MMP-2) increased, while levels of matrix metalloproteinase 9 (MMP-9) decreased in microglia with the LRRK2(R1441G) mutation. Increased proteolytic cleavage of MMP substrates, namely intercellular adhesion molecule 5 (ICAM-5) and alpha-synuclein in synaptoneurosomes from the brain of mice with the LRRK2(R1441G) mutation indicates increased net synaptic MMP activity. PGRN levels were decreased in the CSF of presymptomatic mice with the LRRK2 mutation, while PGRN levels were increased in symptomatic aging mice with the mutation. Interestingly, CSF PGRN levels were elevated in PD patients with LRRK2 mutations but not in idiopathic PD or healthy controls [109]. The decrease in PGRN levels in models carrying LRRK2 mutations and the reciprocal increase in pro-inflammatory cytokines tie PGRN deficits to a known genetic cause of PD. These findings support the hypothesis that PGRN is involved in the molecular foundations of PD, particularly in familial forms or mutation carriers.

The average GRN concentration in patients with PD was significantly lower than in healthy controls [110]. The minor G allele at rs646776 was associated with lower serum GRN levels. Age, sex, and the rs5848 polymorphism did not influence GRN levels in serum. Among AD, amyotrophic lateral sclerosis (ALS), and PD, the strongest association with genomic variation at the GRN gene was observed in PD [111]. A study on a mouse model indicated that PGRN levels decreased during PD, suggesting dysfunction in lysosomal and autophagy pathways. Alpha-synuclein contributed to the downregulation of PGRN at both the mRNA and protein levels in mouse microglial neuroinflammation [111]. It was reported that PGRN levels were lower in patients without dyskinesia compared to healthy controls [16]. However, no significant difference was detected between the dyskinetic and non-dyskinetic groups. Interestingly, in the dyskinetic group, a correlation was detected between PGRN and fibrinogen.

In mice with genetically modified substantia nigra, Parkinsonism was induced using MPTP [112]. Mice modified with vectored PGRN did not show motor slowdown or reduced locomotor activity after MPTP treatment. Neurons in the nigrostriatal pathway were protected from the lethal effects of MPTP following the administration of PGRN via a virus vector. The modification also protected cells from decreased dopamine levels induced by MPTP in the striatum, which is directly associated with preservation of locomotor abilities in this PD model. PGRN protected against the induction of caspase-3 activity and the pyknosis of cells, which leads to apoptosis following MPTP exposure [112]. Moreover, intense microglial activation observed following MPTP exposure was significantly attenuated by PGRN treatment in both the striatum and the substantia nigra. Interestingly, the supply of PGRN did not alter MPTP metabolism to MPP+, suggesting that PGRN does not protect against MPTP toxicity by altering its metabolism. Instead, other mechanisms of PGRN protective action should be considered [113]. It has been demonstrated that serum PGRN levels in PD patients were significantly lower than those in controls [16]. Furthermore, PGRN levels were inversely correlated with the HY score, indicating disease progression. Similarly, serum PGRN levels were negatively correlated with UPDRS I—IV scores and with results on the psychosomatic symptoms questionnaire (PSQ-39). A negative correlation between serum PGRN and disease duration, but not with age, was also observed in the PD patient group [114]. The body of evidence suggests a biologically plausible and functionally significant role for progranulin in PD, particularly in modulating neuroinflammation and microglial activity. Table 5 summarizes human and animal studies on progranulin in PD. However, human studies remain inconclusive due to genetic variability, disease heterogeneity, and the challenges of establishing causality. A more nuanced understanding of the PGRN–GRN axis and its interplay with PD genetics and lysosomal function is needed to translate these insights into therapeutic advances.

In summary, PGRN is a lysosomal growth factor with anti-inflammatory and neurotrophic properties. Reduced PGRN expression has been observed in PD patients, correlating with disease severity and motor impairment. Experimental data confirm its protective role against α-synuclein toxicity and mitochondrial dysfunction. Among adipokines, PGRN currently shows the most consistent link with PD pathophysiology and represents a promising candidate for biomarker development and therapeutic exploration.

## 4. Targeting Adipokines in the Treatment of Parkinson’s Disease

Based on our literature review, we identified several significant challenges in targeting adipokines for PD treatment. Adipokines exhibit pleiotropic, context-dependent effects, functioning as either pro- or anti-inflammatory mediators depending on tissue type and disease stage.

Adipokines, such as leptin and adiponectin, exhibit dual neuroprotective and pro-inflammatory roles. Leptin mitigates oxidative stress but may exacerbate neuroinflammation by activating microglia and elevating TNF-α levels in PD patients [16,32]. Similarly, adiponectin’s anti-inflammatory properties are counterbalanced by its poor BBB penetration, limiting its therapeutic utility despite preclinical promise [16,115]. This duality complicates targeted modulation, as interventions risk unintended consequences. Adipokines interact with broader inflammatory networks, amplifying off-target risks. Among other things, the overlap between leptin, insulin, and erythropoietin signaling pathways raises concerns about metabolic disruptions, such as insulin resistance [115].

Most adipokines act peripherally, requiring innovative CNS-targeted delivery. Although intranasal adiponectin-derived peptides have shown preclinical efficacy in α-synucleinopathy models, their translational potential in humans remains unclear [115]. Systemic leptin modulation may disrupt energy balance, highlighting the need for targeted CNS delivery strategies [16].

Furthermore, the heterogeneity of PD pathophysiology means that only specific subgroups of patients with pronounced inflammatory or metabolic abnormalities may benefit from adipokine-targeted therapies, limiting their overall utility. Adipokine levels fluctuate with PD progression. Patients with dyskinesia show elevated IL-6 and reduced visfatin levels compared to non-dyskinesia groups, while TNF-α remains consistently elevated across PD stages [16]. Such variability suggests that therapies effective in early PD (e.g., targeting leptin) may fail in advanced stages, necessitating dynamic, stage-specific approaches.

Human studies report inconsistent adipokine profiles, partly due to comorbidities (e.g., obesity) and gender differences. Rocha et al. [32] found no significant differences in adiponectin, leptin, or resistin levels between PD patients and controls, questioning the centrality of these factors in PD-associated inflammation. These discrepancies highlight gaps in understanding the links between adipokines and pathology, and hinder the design of clinical trials. Most current studies are cross-sectional and lack mechanistic insight, leaving the molecular pathways through which adipokines might influence neurodegeneration undefined. Thus, while adipokines represent promising links between inflammation and PD progression, their therapeutic potential remains limited without deeper mechanistic understanding, biomarker development, or patient stratification.

## 5. Conclusions

Adipokines, including adiponectin, leptin, progranulin, resistin, and visfatin, play significant roles in the pathophysiology of PD by mediating inflammation, oxidative stress, and metabolic regulation. These molecules may serve as biomarkers of PD progression and severity and, potentially, as therapeutic targets to slow disease progression or alleviate symptoms.

While adipokines show promising neuroprotective effects, their clinical significance in PD remains under investigation. Their potential as biomarkers or therapeutic targets in PD warrants further exploration, particularly concerning their role in inflammation, oxidative stress, and neurodegeneration. Nevertheless, understanding the roles of the described adipokines opens the door to the development of biomarkers for early diagnosis and monitoring progression in PD. Targeting adipokine pathways may offer novel therapeutic strategies to address neurodegeneration, neuroinflammation, and PD-related metabolic disturbances.

## Figures and Tables

**Table 1:** Human, in vitro, and animal studies on leptin in Parkinson’s disease (PD).

Study Characteristics	Results	References
27 PD subjects: 11 weight loss 16 without weight loss 12 control subjects	BMI and fat tissue content positively correlated with leptin levels in PD patients. No correlation was observed between leptin and GH or IGF-1.	Fiszer et al. [23]
23 PD subjects after STN-DBS	Weight gain is associated with improvements in tremor and bradykinesia during the off-medication phase, with a significant correlation observed between changes in leptin levels and these improvements.	Markaki et al. [24]
35 PD subjects 51 control subjects	Statistically insignificant lower leptin levels were observed in PD patients compared to healthy controls. Men showed lower levels than women. A correlation was found between disease duration and BMI in PD patients and controls. UPDRS I: A significant correlation was observed with leptin in both genders. UPDRS II: Correlation with leptin only in women. In men, leptin was negatively correlated with disease duration. Overall, patients with lower BMI tended to have higher UPDRS scores.	Salari et al. [18]
30 PD subjects 30 control subjects	Leptin levels were similar in patients with PD compared to controls. Leptin levels correlated with weight, waist circumference, and BMI in PD, but not with disease duration or dopamine dosage.	Kenangil et al. [33]
36 PD subjects: 18 with unintended weight loss 18 with stable weight	Lower leptin levels were observed in PD patients with documented weight loss, despite no significant differences in appetite, hunger, fullness, food intake, and thirst before meals.	Evidente et al. [35]
26 PD subjects: 14 newly diagnosed, untreated patients 12 advanced PD patients 26 controls	Correlation between leptin and body fat mass in both PD patients and controls. Patients who lost weight had lower leptin levels than those who did not.	Lorefält et al. [31]
40 PD subjects 25 controls	Significant gender differences in leptin levels in PD, but no correlation with clinical indicators of PD.	Ozdilek et al. [37]
55 PD subjects 25 controls	Leptin correlated with BMI and orthostatic systolic blood pressure changes in patients with PD.	Nakamura et al. [38]
44 PD subjects: 20 No dyskinesia 24 With dyskinesia 20 control subjects	Leptin levels were significantly lower in PD patients than in the control group, with the lowest values in patients without dyskinesia.	Milanowski et al. [15]
40 PD subjects 25 control subjects	In PD, higher leptin levels correlated with older age and higher BMI.	Rocha et al. [32]
37 PD subjects with advanced PD undergoing bilateral STN-DBS.	Leptin increased significantly over 6 months post-surgery. At study end, leptin correlated positively with neuropeptide Y in surgically treated patients. In the non-STN-DBS group, neuropeptide Y levels correlated negatively with changes in leptin.	Escamilla-Sevilla et al. [29]
54 PD subjects 55 controls	Leptin correlated significantly with total body fat content, whereas changes in body mass correlated with PD duration.	Bernhardt et al. [34]
47 PD subjects: 15 bilateral DBS 16 on L-DOPA treatment 16 no L-DOPA treatment 16 controls	Leptin was reduced in PD and increased after DBS, reaching control levels.	Carrillo et al. [30]
MN9D cells treated with 6-OHDA.	Leptin reversed 6-OHDA-induced cell death in a dose-dependent manner. No protection was observed after JAK2 and GRB2 knockdown.	Weng et al. [40]
SH-SY5Y cell line treated with MPP+	Leptin induced cell proliferation, counteracting the decrease caused by MPP+. Leptin also preserved mitochondrial membrane potential and ATP levels, while increasing the expression of UCP2 and UCP4.	Ho et al. [48]
Adult male rats exposed to leptin and H_2_S.	6-OHDA reduced leptin production in the rat substantia nigra, and H_2_S reversed this effect. Blocking the leptin signaling pathway reversed the H2S-mediated protective effect against 6-OHDA-induced disruption of the Warburg effect in the substantia nigra.	Yang et al. [20]
Rats treated with 6-OHDA, NaHS, and Leptin-OBR.	NaHS increased leptin expression after prior 6-OHDA administration in the rat substantia nigra. Leptin-OBR antibody administration reversed the effects of NaHS, resulting in reduced caspase-3 activity, increased Bax expression, and decreased Bcl-2 expression. Leptin-OBR administration also reduced the number of autophagosomes and increased the number of autolysosomes in rats treated with 6-OHDA.	Jiang et al. [42]
Male WT rats and LRRK2 exon 41-KO mice	Leptin levels in KO mice on a high-fat diet were slightly lower than in WT mice, but this difference was not statistically significant.	Kawakami et al. [46]
T53A SNCA mutant and WT male mice assessed for leptin levels.	Mice with the T53A mutation in the human SNCA gene had lower leptin levels compared to WT mice. After 12 weeks of a high-calorie diet, leptin levels increased significantly in WT mice but remained substantially lower in SNCA mice under the same dietary conditions.	Rothman et al. [47]

Abbreviations: 6-OHDA—6-Hydroxydopamine, ATP—Adenosine 5′-Triphosphate, Bax—Bcl-2-like Protein 4, Bcl-2—B-cell Lymphoma 2 protein, GH—Growth Hormone, GRB2—Growth Factor Receptor-Bound Protein 2, IGF-1—Insulin-like Growth Factor 1, JAK2—Janus Kinase 2, Leptin-OBR—Leptin-Oncogene Receptor, LRRK2—Leucine-Rich Repeat Kinase 2, MPP+—1-Methyl-4-phenylpyridinium, STN—Subthalamic Nucleus, T53A SNCA—Threonine 53 Alanine Alpha-Synuclein Mutation, UPDRS—Unified Parkinson’s Disease Rating Scale.

**Table 3:** Human and animal studies on resistin in Parkinson’s disease (PD).

Study Characteristics	Results	References
44 PD subjects: -20 No dyskinesia -24 With dyskinesia 20 control subjects	Higher levels of resistin were observed in patients with dyskinesia than in those without. A positive correlation with the Hoehn and Yale scale.	Milanowski et al. [15]
40 PD subjects 25 control subjects	No significant changes detected.	Rocha et al. [32]
C57/BL6 mice injected with resistin into the lateral ventricles of the brain, followed by immunofluorescence analysis of brain tissue. HBMEC cells incubated with resistin.	A decrease in the number of GFAP+/BrdU+ cells after resistin treatment indicates that resistin may inhibit astrocyte differentiation.	Xiaoying et al. [83]
MES23.5 cells.	Resistin reversed the effects of 6-OHDA on pro-apoptotic properties, reduced intracellular ROS, protected against the decline in mitochondrial membrane potential, decreased levels of caspase 3 and PARP-1/2, and induced an increase in Hsc73.	Lu et al. [84]

Abbreviations: 6-OHDA—6-hydroxydopamine, GFAP+/BRdU+ cells—glial fibrillary acidic protein/bromodeoxyuridine positive cells, HBMEC cells—human brain microvascular endothelial cells, Hsc73—heat shock cognate 71 kDa protein, PD—Parkinson’s disease, ROS—reactive oxygen species.

**Table 4:** Human studies on visfatin in Parkinson’s disease (PD).

Study Characteristics	Results	References
18 patients undergoing DBS surgery: -11 PD subjects -7 ET subjects	NAMPT and NADH increased significantly in PD patients.	Ostrakhovitch et al. [92]
52 PD subjects: -26 no dyskinesia -26 with dyskinesia 26 control subjects	NAMPT correlated with fibrinogen; NAMPT levels were significantly higher in the group without dyskinesia than in the group with dyskinesia.	Milanowski et al. [16]
Post-mortem examination of NAMPT and NMNAT in the caudate nucleus vs. monomeric alpha-synuclein	FK866, an inhibitor of NAMPT, may support neurons against the expression of alpha-synuclein in DA neuron-like SH-SY5Y cells	Parsons et al. [93]
mRNA NAMPT evaluated in PD patients	mRNA NAMPT 69% accurate in distinguishing progressive supranuclear palsy from healthy controls; no such conclusions drawn for PD and atypical Parkinsonian disorders.	Santiago et al. [94]

Abbreviations: DBS—deep brain stimulation, FK866—nicotinamide phosphoribosyltransferase inhibitor, NADH—nicotinamide adenine dinucleotide, NAMPT—nicotinamide phosphoribosyltransferase, PD—Parkinson’s disease, SH-SY5Y cells—human neuroblastoma cells.

**Table 5:** Human and animal studies on progranulin in Parkinson’s disease (PD).

Study Characteristics	Results	References
771 PD subjects 642 controls	No correlation found between the GRN rs5848 T allele and PD in US, Polish, and combined datasets. The rs5848 TT genotype occurrence is comparable between PD patients and controls.	Jasinska-Myga et al. [101]
573 PD subjects 490 controls	The GRN rs5848 SNP was found to affect PD susceptibility in Taiwanese individuals. A higher prevalence was observed in individuals with the TT genotype, particularly among females.	Chang et al. [105]
1270 PD subjects 830 controls	The minor allele T of the GRN rs5848 gene is less common in patients with PD than in controls. The GRN rs5458 variant is associated with a reduced risk of PD in the Chinese population.	Chen et al. [106]
255 PD subjects 459 controls	In the Belgian population, no association was found between PD and mutations in the PGRN gene.	Nuytemans et al. [102]
Data from the three most extensive publicly available studies on AD, PD, and ALS for the GRN gene.	The strongest association with genomic variation at the GRN gene was observed in PD, compared to AD and ALS.	Nalls et al. [111]
7 PD subjects	Postural tremor associated with GRN mutations in PD.	Saracino et al. [107]
43 PD subjects	Serum GRN levels are lower in Belgian patients. No correlation between age at symptom onset and GRN levels.	Wauters et al. [103]
52 PD subjects: -26 No dyskinesia -26 With dyskinesia 26 control subjects	Lower PGRN levels were found in patients without dyskinesia compared to healthy controls. A correlation was detected between PGRN and fibrinogen in the dyskinetic group.	Milanowski et al. [16]
63 FTLD subjects	Intrafamilial clinical heterogeneity observed. Different diagnoses given within the family; Parkinsonism in the course of FTLD or PD has not been ruled out. Cortico-subcortical atrophy ipsilateral to the dominant Parkinsonian syndrome was detected post-mortem.	Rovelet-Lecrux et al. [108]
55 PD subjects 55 controls	PGRN levels are significantly lower than in controls. PGRN levels correlated with the HY score and negatively correlated with UPDRS I-IV and PSQ-39. A negative correlation was observed with disease duration, but not with age.	Yao et al. [114]
304 PD subjects 126 controls	The average GRN is significantly lower than in healthy controls. Minor G allele in the rs646776 polymorphism associated with lower serum GRN levels. Age, sex, and rs5848 polymorphism did not influence GRN levels in serum in any subgroup.	Mateo et al. [110]
255 PD subjects	The clinical diagnosis of PD in individuals with null mutations in PGRN is likely due to etiological heterogeneity rather than PGRN haploinsufficiency.	Brouwers et al. [104]
9 LRRK2(G2019S) mutation carriers 9 controls	PGRN reduced in mouse fibroblasts (LRRK2(R1441G)) and human fibroblasts (LRRK2(G2019S)). Increased pro-inflammatory factors (IL1B, keratinocyte-derived chemokine) were observed. MMP-2 increased, MMP-9 decreased in microglia (LRRK2(R1441G)). Increased synaptic MMP activity noted. PGRN levels decreased in presymptomatic mice with LRRK2 mutation, increased in symptomatic aging mice, and in PD patients with LRRK2 mutations.	Caesar et al. [109]
PCR, immunohistochemistry, and functional studies were conducted on a mouse microglial cell model.	Progranulin levels decreased during PD progression, indicating dysfunction in lysosomal and autophagy pathways. αSyn is implicated in reducing PGRN levels at both mRNA and protein levels in mouse microglial neuroinflammation.	Kanthasamy et al. [112]
Mice with lentiviral-modified substantia nigra, MPTP-induced Parkinsonism.	PGRN prevented motor slowdown and reduced locomotor activity after MPTP treatment, protected neurons in the nigrostriatal pathway from MPTP-induced damage, preserved dopamine levels in the striatum, inhibited caspase-3 activity and apoptosis induction, and attenuated microglial activation in the striatum and substantia nigra.	Kampen et al. [113]

Abbreviations: LRRK2—leucine-rich repeat kinase 2, MMP—matrix metalloproteinase, PD—Parkinson’s disease, UPDRS—Unified Parkinson’s Disease Rating Scale.

## Data Availability

No new data were created or analyzed in this study. Data sharing is not applicable to this article.

## Abbreviations

6-OHDA	6-Hydroxydopamine
∆Ψm	Mitochondrial Membrane Potential
ADI	Adiponectin
AdipoR1	Adiponectin Receptor 1
AdipoR2	Adiponectin Receptor 2
AMPK	AMP-Activated Protein Kinase
ATP	Adenosine Triphosphate
Bax	Bcl-2-like Protein 4
BBB	Blood–Brain Barrier
Bcl-2	B-cell Lymphoma 2 Protein
BMI	Body Mass Index
COMT	Catechol O-Methyltransferase
DBS	Deep Brain Stimulation
DJ-1	Parkinson Disease Protein 7
FK866	Nicotinamide Phosphoribosyltransferase Inhibitor
GFAP+/BrdU+	Glial Fibrillary Acidic Protein/Bromodeoxyuridine Positive Cells
GH	Growth Hormone
GPi	Globus Pallidus Internus
GRB2	Growth Factor Receptor-Bound Protein 2
HBMEC	Human Brain Microvascular Endothelial Cells
HDL	High-Density Lipoprotein
HO-1	Heme Oxygenase 1
Hsc73	Heat Shock Cognate 71 kDa Protein
ICAM-5	Intercellular Adhesion Molecule 5
IGF-1	Insulin-Like Growth Factor 1
IL-6	Interleukin 6
IL-8	Interleukin 8
JAK2	Janus Kinase 2
LDH	Lactate Dehydrogenase
Leptin-OBR	Leptin-Oncogene Receptor
LRRK2	Leucine-Rich Repeat Kinase 2
L166P	Leucine 166 Proline Mutation
MCP-1	Monocyte Chemoattractant Protein 1
MCF-7	Michigan Cancer Foundation-7 Cell Line
MMP	Matrix Metalloproteinase
MMP-2	Matrix Metalloproteinase 2
MPP+	1-Methyl-4-Phenylpyridinium
MPTP	1-Methyl-4-Phenyl-1,2,3,6-Tetrahydropyridine
MTP assay	Mitochondrial Transmembrane Potential Assay
MTT assay	3-(4,5-Dimethylthiazol-2-yl)-2,5-Diphenyltetrazolium Bromide Assay
NAD+	Nicotinamide Adenine Dinucleotide (Oxidized Form)
NADH	Nicotinamide Adenine Dinucleotide (Reduced Form)
NAMPT	Nicotinamide Phosphoribosyltransferase
NMNAT	Nicotinamide Mononucleotide Adenylyltransferase
NO	Nitric Oxide
PARP-1/2	Poly(ADP-Ribose) Polymerase 1/2
PD	Parkinson’s Disease
PGRN	Progranulin
PPARγ	Peroxisome Proliferator-Activated Receptor Gamma
ROS	Reactive Oxygen Species
RT-PCR	Reverse Transcription Polymerase Chain Reaction
SH-SY5Y	Human Neuroblastoma Cells
SNCA	Alpha-Synuclein
SOCS3	Suppressor of Cytokine Signaling 3
SOD	Superoxide Dismutase
SRB assay	Sulforhodamine B Assay
STN	Subthalamic Nucleus
T53A SNCA	Threonine 53 to Alanine Alpha-Synuclein Mutation
TNF	Tumor Necrosis Factor
TNF-α	Tumor Necrosis Factor Alpha
UPDRS	Unified Parkinson’s Disease Rating Scale
WT	Wild-Type
